# Sarcopenia and Myosteatosis as Prognostic Markers in Patients with Advanced Cholangiocarcinoma Undergoing Palliative Treatment

**DOI:** 10.3390/jcm10194340

**Published:** 2021-09-23

**Authors:** Markus S. Jördens, Linda Wittig, Lisa Heinrichs, Verena Keitel, Maximilian Schulze-Hagen, Gerald Antoch, Wolfram T. Knoefel, Georg Fluegen, Tom Luedde, Christina Loberg, Christoph Roderburg, Sven H. Loosen

**Affiliations:** 1 Department of Gastroenterology, Hepatology and Infectious Diseases, University Hospital Düsseldorf, Medical Faculty of Heinrich Heine University Düsseldorf, 40225 Düsseldorf, Germany; linda.wittig@med.uni-duesseldorf.de (L.W.); lisa.heinrichs@med.uni-duesseldorf.de (L.H.); Verena.Keitel@med.uni-duesseldorf.de (V.K.); Tom.Luedde@med.uni-duesseldorf.de (T.L.); Christoph.Roderburg@med.uni-duesseldorf.de (C.R.); 2 Department for Diagnostic and Interventional Radiology, University Hospital Aachen, 52074 Aachen, Germany; mschulze@ukaachen.de; 3 Department of Diagnostic and Interventional Radiology, University Hospital Düsseldorf, Medical Faculty of Heinrich Heine University Düsseldorf, 40225 Düsseldorf, Germany; gerald.antoch@med.uni-duesseldorf.de (G.A.); christina.loberg@med.uni-duesseldorf.de (C.L.); 4 Department of General, Visceral and Pediatric Surgery, University Hospital Düsseldorf, Medical Faculty of Heinrich Heine University Düsseldorf, 40225 Düsseldorf, Germany; knoefel@med.uni-duesseldorf.de (W.T.K.); Georg.Fluegen@med.uni-duesseldorf.de (G.F.)

**Keywords:** cholangiocellular carcinoma, CCA, sarcopenia, myosteatosis, MMA, prognostic marker, survival

## Abstract

Background: Cholangiocarcinoma (CCA) represents the second most common primary liver cancer and is characterized by a very poor outcome, but reliable prognostic markers are largely missing. Sarcopenia, the progressive loss of muscle mass and strength, as well as myosteatosis have been associated with an unfavorable outcome in several clinical conditions, including cancer. Here, we evaluated the prognostic relevance of sarcopenia and myosteatosis using routine abdominal CT (computed tomography) scans in advanced stage CCA patients undergoing palliative treatment. Methods: Routine abdominal CT scans were used to assess the skeletal muscle and the psoas muscle index (L3SMI/L3PMI) at the level of the third lumbar vertebra as radiological indices for sarcopenia as well as the mean skeletal muscle attenuation (MMA) as a surrogate for myosteatosis. Results were correlated with clinical data and outcomes. Results: Using a calculated optimal cut-off value of 71.95 mm^2^/cm, CCA patients with an L3SMI value below this cut-off showed a significantly reduced median overall survival (OS) of only 250 days compared to 450 days in patients with a higher L3SMI. Moreover, the median OS of CCA patients with an L3PMI above 6345 mm^2^/cm was 552 days compared to 252 days in patients with a lower L3PMI. Finally, CCA patients with an MMA above 30.51 Hounsfield Units survived significantly longer (median OS: 430 days) compared to patients with an MMA value below this ideal cut-off (median OS: 215 days). The prognostic relevance of L3SMI, L3PMI, and MMA was confirmed in uni- and multivariate Cox regression analyses. Conclusion: Routine abdominal CT scans represent a unique opportunity to evaluate sarcopenia as well as myosteatosis in advanced CCA patients. We identified the L3SMI/L3PMI as well as the MMA as negative prognostic factors in CCA patients undergoing palliative therapy, arguing that the “opportunistic” evaluation of these parameters might yield important clinical information in daily routine.

## 1. Introduction

Cholangiocarcinoma (CCA) is the second most common primary malignancy of the liver and is characterized by a very poor outcome [1]. While surgical tumor resection represents a potentially curative therapeutic option in patients with early tumor stages, patients with locally advanced or metastatic CCA are usually attributed to a palliative treatment approach including systemic chemotherapy or active symptom control (ASC) in case of a poor clinical performance status [2]. The combination of gemcitabine and cisplatin has been established as the standard first-line therapy for unresectable CCA patients and significantly improves the median overall survival (OS) to 11.7 months [3]. Following progression to gemcitabine and cisplatin, the addition of FOLFOX (folinic acid, fluorouracil, and oxaliplatin) to ASC has been shown to improved median OS to 6.2 months compared to 5.3 months in the ASC-alone group [4]. However, treatment response and outcome to first and/or second-line chemotherapy is often heterogeneous, and prediction of the individual patients’ prognosis has remained a challenge in clinical routine [5]. Especially in view of the fact that systemic therapy can be associated with significant side effects [3,4] and predictive biomarkers are mainly experimental [6], the question arises as to which patients represent the ideal therapy candidates.

The term “sarcopenia” has recently been defined by the international working group on sarcopenia as the “progressive loss of muscle mass and strength with a risk of adverse outcomes such as disability, poor quality of life and death” [7]. In cancer patients, sarcopenia is most likely caused by systemic inflammatory processes that lead to catabolic nutrition state, a decreased appetite, as well as immobility [8], and it has previously been associated with an impaired outcome [9]. In addition, an increasing deterioration of muscle quality in the sense of myosteatosis is a frequently observed phenomenon in cancer patients, which is associated with an unfavorable prognosis [10]. As most cancer patients, including CCA patients, receive CT (computed tomography) scans to evaluate the tumor stage and response to therapy on a regular basis, these existing imaging data represent a unique opportunity to systematically assess sarcopenia and myosteatosis without further costs or burden on the patients. 

Therefore, in the present study, we used routine CT scans to determine the skeletal muscle index (L3SMI) and the psoas muscle index (L3PMI) at the level of the third lumbar vertebra as radiological indices for sarcopenia as well as the mean skeletal muscle attenuation (MMA) as a surrogate for myosteatosis in order to evaluate its prognostic relevance in CCA patients undergoing palliative treatment.

## 2. Patients and Methods

### 2.1. Selection of Study Patients

We included 75 patients with CCA in palliative treatment in our study. All patients were treated at the Department of Gastroenterology, Hepatology and infectious diseases or the Department for General, Visceral, and Pediatric Surgery at the University Hospital Düsseldorf between 2011 and 2021 (Detailed patients’ characteristics are presented in Table 1). In total, 64 patients underwent palliative systemic chemotherapy, and 11 patients received best supportive care. Fifty of the 75 patients died during the observation period, and 25 were lost to follow-up. Of all the included patients, we used CT scans performed before the start of chemotherapeutic treatment or best supportive care. Progressive disease was diagnosed by an experienced radiologist based on the radiological CT findings in restaging. In this context, the current RECIST criteria were applied. The study was approved by the ethics committee of the medical faculty of Heinrich Heine University Düsseldorf (2021-1334).

### 2.2. Analysis of Sarcopenia

**Figure 1:**
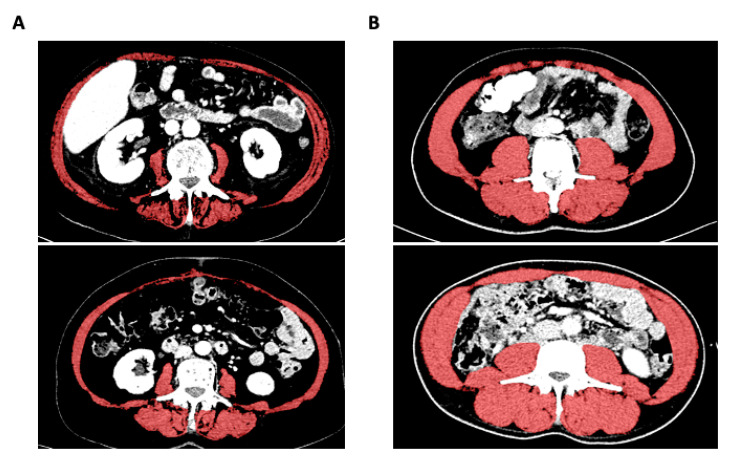
Evaluation of the L3SMI using the 3D slicer tool. Exemplary presentation of CT scans from patients with low (**A**) or high (**B**) L3SMI value.

We used the venous phase of CT scans obtained before the start of systemic therapy or best supportive care to analyze sarcopenia. For L3SMI, we measured the areas of the m. psoas, m. erector spinae, m. quadratus lumborum, m. rectus abdominis, m. transversus abdominis, m. obliquus abdominis internus, and m. obliquus abdominis externus at the level of the third lumbar vertebra in a single slice with a semi-automatic segmentation tool (*3D Slicer*, [11]). To determine the psoas muscle index at the level of the 3rd lumbar vertebra (L3PMI), only the left m. psoas was measured (Figure 1). We quantified muscles with attenuation values between −29 and 150 Hounsfield Units (HU). The *3D Slicer* software calculates the mean skeletal muscle attenuation (MMA) and the muscle area as an absolute value. MMA is defined as the mean muscle density, which directly correlates with the amount of fat deposition within the muscle. L3SMI is defined as the total muscle area at the level of the third lumbar vertebra normalized for patients´ height. The L3PMI is defined as the area of the left psoas muscle at the level of the third lumbar vertebra normalized for the patients’ height [12]. 

### 2.3. Statistical Analysis

All statistical analyses were performed using SPSS 27 (SPSS, Chicago, IL, USA) unless otherwise described. Tests were similar performed as described in detail before [12]. To determine the normal distribution, a Shapiro-Wilk test was used. The Spearman correlation was performed for all correlation analyses. Using a Mann-Whitney U-test (two values) or Kruskal-Wallis test (more than two values), non-parametric data were compared. Medians, quartiles, and ranges are indicated in the shown box plots. Kaplan–Meier curves were generated to analyze the influence of different parameters on overall survival, and statistical differences between groups were tested with the log-rank test. To determine the optimal cut-off values for L3SMI, L3PMI, and MMA, we used the optimal cut-off finder, as described before [13]. Furthermore, we performed univariate and multivariate Cox regression analyses to analyze the prognostic value of different variables with regard to overall survival. The hazard ratio (HR) and 95% confidence interval (CI 95%) are displayed. *p*-values less than 0.05 are considered statistically significant.

## 3. Results

### 3.1. Baseline Characteristics of Patients

In this study 75 patients were included, of which 53.3% were male and 46.7% were female (Table 1). The median age was 70 years (range 30–87), and the median BMI (body mass index) was 24.2 (18.5–44.3). Most (85.3%) of the total patients had received palliative systemic chemotherapy as follows: Gemcitabine and Cisplatin 81.3%, Gemcitabine and Oxaliplatin 4.7%, Carboplatin and Paclitaxel 1.6%, Capecitabine 1.6%, Gemcitabine 10.7%. More than one-quarter (28%) of patients had progressive disease at 6 months follow up, while 69% had metastatic disease, and the localization of metastases was as follows: lymphatic 20%, vascular infiltration 8%, pulmonary 18.7%, bone 10.7%, suprarenal gland 1.3%, peritoneal 22.7%, other 16%. Pre-existing medical conditions were as follows: preceded tumor disease 24%, preceded systemic chemotherapy 1.3%, diabetes mellitus type 2 29.3%, arterial hypertension 58.7%, hepatitis B 6.7%, hepatitis C 5.3%, abuse of alcohol 1.3%, primary biliary cholangitis 2.7%, primary sclerosing cholangitis 1.3%, non-alcoholic steatohepatitis 2.7%, inflammatory bowel disease 1.3%, gastritis 24%. The median overall survival of the study cohort was 224 (3–1059) days. Median progression-free survival was 132 (3–916) days. No difference between female or male sex regarding overall survival was identified (*p* = 0.644; Figure S1), and the presence of metastases had also no significant influence on overall survival (*p* = 0.106; Figure S2). 

### 3.2. L3SMI Is Dependent on Patients’ Sex but Independent of Age, Albumin, or CRP Levels

**Figure 2:**
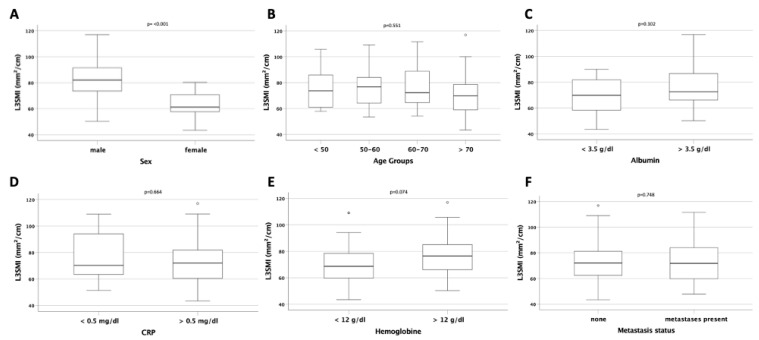
Evaluation of the L3SMI in different patient subgroups. (**A**) Male patients have a significantly higher L3SMI compared to female patients. The L3SMI is unaltered between patients of different age (**B**), with normal or reduced serum albumin levels (**C**) and normal or increased CRP serum levels (**D**). (**E**) Anemic patients show a trend toward a reduced L3SMI compared to non-anemic patients. The L3SMI is unaltered between patients with and without distant metastases (**F**).

As expected, L3SMI before palliative therapy was higher in men compared to women (*p* < 0.001; Figure 2A). There was no significant difference of the L3SMI between younger and older patients or between patients with a reduced or normal albumin serum concentration as a marker for malnutrition (*p* = 0.551 and *p* = 0.102; Figure 2B,C). To analyze the potential impact of inflammation on L3SMI, we compared patients with elevated CRP serum concentration with patients with normal CRP serum levels. However, CRP serum concentration had no effect on the L3SMI (*p* = 0.664; Figure 2D). We compared patients with normal or reduced hemoglobin serum levels as a marker for anemia and identified a clear trend toward a better L3SMI in non-anemic patients (*p* = 0.074; Figure 2E). Finally, we could not identify a significant difference of the L3SMI between patients with or without metastases as a marker for advanced disease stage (*p* = 0.748; Figure 2F).

### 3.3. L3PMI Is Dependent on Sex and Albumin Serum Concentration but Independent of Age and CPR

**Figure 3:**
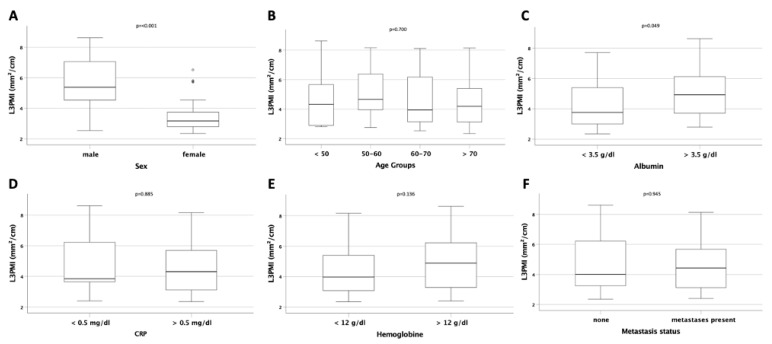
Evaluation of the L3PMI in different patient subgroups. (**A**) Male patients have a significantly higher L3PMI compared to female patients. The LPSMI is unaltered between patients of different age (**B**). (**C**) Patients with reduced serum albumin levels have a significantly lower LPSMI compared to patients with normal serum albumin levels. The L3PMI is unaltered between patients with normal or increased CRP serum levels (**D**), anemic or non-anemic patients (**E**), or patient with and without distant metastases (**F**).

The L3PMI was also higher in male patients compared to female patients (*p* < 0.001; Figure 3A), and there was no difference between different age groups or between patients with normal or elevated CRP serum concentrations (*p* = 0.700 and *p* = 0.885; Figure 3B,D). In contrast to the L3SMI, we could identify a significantly higher L3PMI in patients with albumin serum concentrations above 3.5 g/dL (*p* = 0.049; Figure 3C). Furthermore, neither serum hemoglobin concentration nor the presence of metastases had an influence on the L3PMI (*p* = 0.136 and *p* = 0.945; Figure 3E,F). 

### 3.4. L3SMI and L3PMI Are Predictors of Overall Survival in CCA Patients under Palliative Therapy

**Figure 4:**
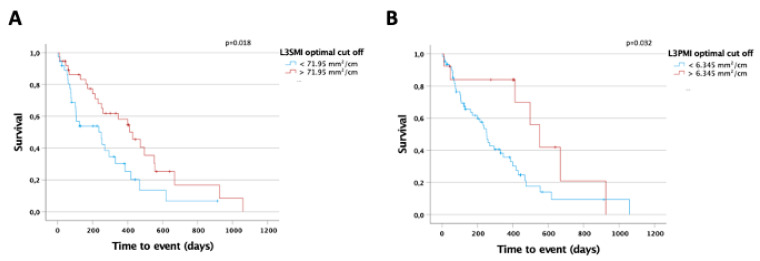
L3SMI and L3PMI are predictors of overall survival in CCA patients under palliative therapy. (**A**) CCA patients with an L3SMI value below the calculated ideal prognostic cut-off value of 71.95 mm^2^/cm show a significantly reduced overall survival compared to patients with an L3SMI above the cut-off. (**B**) CCA patients with an L3PMI value below the calculated ideal prognostic cut-off value of 6.345 mm^2^/cm show a significantly reduced overall survival compared to patients with an L3PMI above the cut-off.

As sarcopenia is known to be a prognostic marker for overall survival (OS) in various malignant and non-malignant clinical conditions, we next evaluated a potential prognostic relevance of the L3SMI and L3PMI on patients’ OS in our cohort of patients. We established ideal prognostic cut-off values using recently published software that identifies the respective L3SMI and L3PMI value with the most significant log-rank test in Kaplan–Meier curve analysis [13]. Using the optimal cut-off value, we could show that patients with an L3SMI value above 71.95 mm^2^/cm lived significantly longer compared to patient with an L3SMI below this value (430 days (95%CI: 298–562) vs. 250 days (95%CI: 76–424) days; log rank χ^2^ (1) = 5.622, *p* = 0.18; Figure 4A). In line, univariate Cox regression analysis revealed that an L3SMI value below the ideal cut-off value is a negative prognostic factor regarding OS (HR 1.990, 95%CI: 1.115–3.551, *p* = 0.020, Table 2). In line with these results, we could also establish a prognostic relevance of the L3PMI. When stratified according to the calculated ideal cut-off value of 6.345 mm^2^/cm, the median OS of patients with an L3PMI above this value was significantly higher compared to patients with an L3PMI below (552 days (95%CI: 413–690) vs. 252 days (95%CI: 217–287); log rank χ^2^ (1) = 4.600, *p* = 0.32; Figure 4B). Again, univariate Cox regression analysis revealed that an L3PMI value below the ideal cut-off value represents a negative prognostic factor for OS (HR 2.384, 95%CI: 1.054–5.393, *p* = 0.037, Table 2).

### 3.5. The MMA Is a Predictor for Overall Survival in Patients with Palliative Treatment for CCA

**Figure 5:**
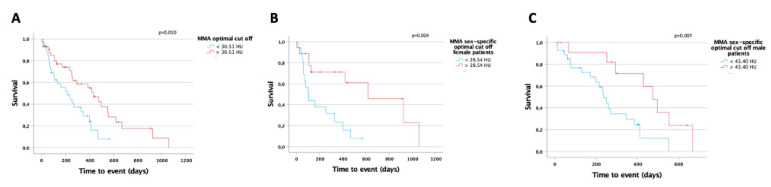
The MMA is a predictor for overall survival in patients with palliative treatment for CCA. (**A**) CCA patients with an MMA above 30.51 Hounsfield Units (HU) live significantly longer compared to patients with a MMA value below this ideal cut-off. Both in female ((**B**), 28.54 HU) and male patients ((**C**), 43.40 HU), the ideal sex-specific cut-off values have a significant prognostic value regarding median OS.

The mean skeletal muscle attenuation (MMA) represents a marker to assess the muscle’s quality (myosteatosis) with lower values indicating a higher lipid concentration within the muscle [14]. We next calculated an optimal MMA cut-off value to evaluate a potential role of the MMA as a prognostic marker for OS in our cohort of patients. CCA patients with an MMA above 30.51 Hounsfield Units (HU) lived significantly longer compared to patients with an MMA value below this ideal cut-off (430 days (95%CI: 234–626) vs. 215 days (95%CI: 88–342); log rank χ^2^ (1) = 6.709, *p* = 0.010; Figure 5A). To identify possible sex-specific differences for the MMA, we performed subgroup analyses and calculated sex-specific cut-off values for the MMA. Both in female (28.54 HU) and male patients (43.40 HU), the ideal sex-specific cut-off values showed a statistically significant prognostic value regarding median OS (female: 619 days (95%CI: 189–1049) vs. 105 days (95%CI: 57–153); log rank χ^2^ (1) = 8.301, *p* = 0.004; male: 474 days (95%CI: 382–566) vs. 237 days (95%CI: 188–286); log rank χ^2^ (1) = 7.207, *p* = 0.007; Figure 5B,C). To further corroborate the prognostic relevance of the MMA, we performed uni- and multivariate Cox regression analyses. In univariate analysis, an MMA value below the ideal cut-off value represented a negative prognostic factor for OS (HR 2.384, 95%CI: 1.054–5.393, *p* = 0.037, Table 2). In multivariate analysis, the prognostic value of the MMA was independent of CRP, thrombocytes, routinely used tumor markers (CEA and CA19-9) as well as of the L3SMI and L3PMI (HR 2.264, 95%CI: 1.059–4.842; *p* = 0.035; Table 3).

### 3.6. The Combination of MMA and L3SMI Is a Strong Predictor for Overall Survival

To further evaluate the prognostic power of muscle mass and quality, we performed Kaplan–Meier analyses and combined L3SMI as a marker for muscle mass and MMA as a marker for muscle quality. Patients with L3SMI and MMA above the before established cut-off values had a significantly better overall survival then patients with L3SMI and MMA under the optimal cut-off values. The overall survival of patients with either L3SMI or MMA over the optimal cut-off had a better survival than patients with both values under the optimal cut-off but worse than patients with both values over the optimal cut-off. This observation strengthens our assertion that both muscle mass and quality are important prognostic markers for overall survival in patients undergoing palliative therapy for CCA and that the combination of both is especially valuable.

## 4. Discussion

Different lines of evidence are available for a role of the body composition as a predictive and/or prognostic marker in patients with gastrointestinal cancers [15,16]. Both experimental and clinical analyses have demonstrated an impaired response to treatment and an impaired overall survival in patients with a reduced muscle mass and/or an impaired muscle quality [17]. Nevertheless, many questions including the magnitude of the associations and whether parameters that have been established in patients receiving surgery can be translated to patients in palliative disease stages have remained unanswered [18]. Here, we demonstrate that routine CT scans can be used to easily assess sarcopenia as well as myosteatosis in patients receiving palliative therapy for advanced-stage CCA and that both parameters represent prognostic markers for OS. These findings suggest that “opportunistic” imaging analyses can yield important information on the individual patient’s characteristics and should therefore be implemented into clinical routine.

**Figure 6:**
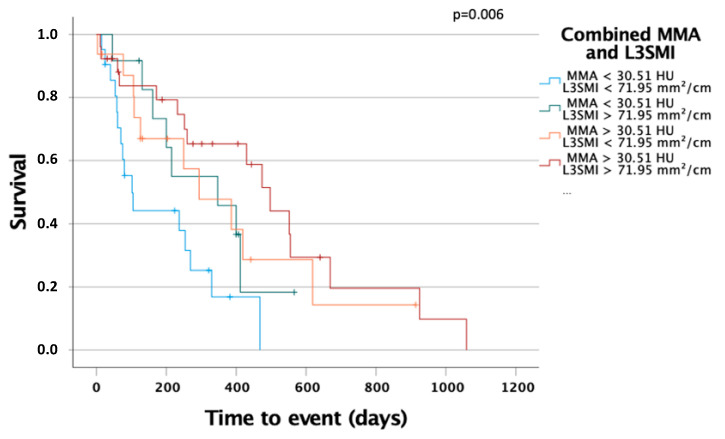
The combination of MMA and L3SMI is a strong predictor for overall survival in patients with palliative treatment for CCA. Patient with MMA above 30.51 HU and L3SMI above 71.95 mm^2^/cm had significantly improved OS compared to patients with both markers under their optimal cut-off or combinations.

The optimal method for body composition analysis has been the subject of intense debate in recent years. Many of the available methods can be biased, for example, by fluid overload, as it frequently occurs during the course of chemotherapy. Therefore, we used baseline CT scans to quantify the area of skeletal muscle as well as the skeletal muscle attenuation as a surrogate for sarcopenia. CT imaging is available for almost all CCA patients before chemotherapy, allowing cost-effective analyses of multiple surrogates for the body composition, as it was recently demonstrated in different clinical contexts [16,19]. Therefore, in contrast to most of the previous studies on biliary tract cancer that relied on measurements of the psoas muscle area only [20,21,22], we are able to provide a more complete picture of the individual body composition by simultaneously measuring muscle mass (L3SMI and L3PMI) and myosteatosis (MMA). So far, the role of sarcopenia and myosteatosis as prognostic factors in biliary tract cancer patients is largely unclear. Within this study, we quantified both the skeletal muscle mass and the amount of skeletal muscle fat deposition and demonstrated that an “impaired” body composition indicates an impaired clinical outcome in patients receiving chemotherapy for biliary tract cancer. At the respective optimal cut-off values that we established using a recently described biometric software [13], both a low L3SMI and L3PMI as well as a low skeletal muscle HU turned out to be powerful predictors of OS. Importantly, when both markers were combined (e.g., SMI low/MMA low vs. SMI high/MMA high patients), the prognostic potential was even further increased (see Figure 6). 

Along with these analyses suggesting a use of L3SMI, L3PMI, and MMA as stand-alone markers for estimating patients’ overall survival, we further tested different combinations of L3SMI/MMA as prognostic markers, revealing that patients with low muscle mass and low muscle quality had the worst prognosis of all patients (Figure 6). Interestingly, these data are in line with a recent analysis by Yoon et al., demonstrating that both reduced muscle mass and muscle quality are indicative for an impaired prognosis of patients following the resection of biliary tract cancer [23]. In the past, reduced muscle mass, e.g., indicated by a reduced L3SMI or L3PMI, was used to assess the presence of sarcopenia. In patients with chronic diseases, low muscle mass is the result of an impaired muscle growth in the setting of an increased muscle wasting. However, it became clear that some patients show a much greater loss of muscle strength than would be expected after a loss of muscle mass. In these patients, increased intramuscular fat deposition has been described, as reflected by a change in MMA in our study [24]. In this context, a recently published study suggested that fatty degeneration of the muscle in tumor patients is an even earlier event; i.e., it occurs before loss of muscle mass [25]. Therefore, it seems likely that different pathophysiological mechanisms underlie these processes. Nevertheless, further clinical and translational studies are needed to fully understand the mechanism driving muscle loss and myosteatosis in cancer patients. 

Sarcopenia is a common characteristic of elderly and moribund patients. Muscle wasting can be triggered by manifold disease conditions including cardiac failure and renal dysfunction [26]. As all of these factors are frequently found in patients with cancer and might themselves limit patients’ prognosis, systematic research on the role of an impaired muscle mass and strength bears a risk of important bias. Nevertheless, in our analysis, we could identify L3SMI, L3PMI, and MMA as predictive factors for OS for patients undergoing palliative treatment for CCA. Especially MMA proved to be an independent and reliable prognostic factor regarding OS in multivariant regression analyses. Nevertheless, further studies are warranted to not only confirm their prognostic role in the context of biliary tract cancer but also to assess if the L3SMI, L3PMI, and the MMA could support future biomarker-driven clinical decision algorithms in the multimodal treatment of cancer. Importantly, our analysis only gave information on the prognosis of these patients but had no predictive value, meaning that it is unclear if those patients with an unfavorable prognosis in terms of their individual L3SMI, L3PMI, or MMA value might have had benefited to a greater extend from other treatments or even represent candidates for active symptom control who should not been administered chemotherapy. Moreover, we only evaluated SMI and MMA values before starting chemotherapy, but longitudinal measurements of SMI and MMA could also be important to answer the question of whether therapeutic interventions affecting body composition might influence patients’ outcome. Furthermore, potential important information on factors influencing overall survival such as biliary stenting were often missing due to the nature of retrospective data and could not be analyzed. Nevertheless, our study demonstrated a potential prognostic value of sarcopenia and myosteatosis in the context of CCA patients. Moreover, our study demonstrated that the simultaneous assessment and combined analysis of different parameters of the patients’ individual body composition might be superior over a single marker for estimating the long-term outcome of these CCA patients. Such data might provide important information for clinical decision making on patients allocated to a systemic chemotherapy for CCA. Furthermore, these data can be used to decide on nutritional therapy in the context of multimodal treatment concepts in CCA patients. Therefore, our data should induce further clinical research but also basic research on the importance of body composition in the context of malignant diseases and CCA in particular.

## Figures and Tables

**Table 1:** Study cohort.

Parameter	Study Cohort
CCA patients	*n* = 75
Sex % (*n*): male female	53.3 (40) 46.7 (35)
Age (years, median and range)	70 (30–87)
BMI Median kg/m^2^ (range)	24.2 (18.5–44.3)
BMI class kg/m^2^% (*n*) BMI < 20 BMI 20–25 BMI 25–30 BMI > 30	9.3 (7) 45.3 (34) 28 (21) 17.3 (13)
Systemic therapy % (*n*) Yes No	85.3 (64) 14.7 (11)
Chemotherapy regimen % (*n*) Gemcitabine + Cisplatin Gemcitabine + Oxaliplatin Carboplatin + Paclitaxel Capecitabine Mono Gemcitabine Mono	81.3 (52) 4.7 (3) 1.6 (1) 1.6 (1) 10.9 (7)
Tumor progression during follow-up? % (*n*) Yes No	28 (21) 72 (54)
Metastatic Disease % (*n*) Yes No	69 (52) 31 (23)
Localization of tumor metastasis % (*n*) Lymphatic Vascular Pulmonary Bone Suprarenal gland Peritoneum Other	20 (15) 8 (6) 18.7 (14) 10.7 (8) 1.3 (1) 22.7 (17) 16 (12)
Pre-existing medical conditions % (*n*) Preceded tumor disease Preceded systemic chemotherapy Diabetes mell. Type 2 Arterial Hypertension Hepatitis B Hepatitis C Alcohol abuse Primary biliary cholangitis Primary sclerosing cholangitis Non-alcoholic steatohepatitis Inflammatory bowel disease Gastritis	24 (18) 1.3 (1) 29.3 (22) 58.7 (44) 6.7 (5) 5.3 (4) 1.3 (1) 2.7 (2) 1.3 (1) 2.7 (2) 1.3 (1) 24 (18)
Overall survival (days, median and range)	224 (3–1059)
Progression-free survival (days, median and range)	132 (3–916)

BMI, body mass index.

**Table 2:** Univariate Cox regression analysis for the prediction of overall survival.

Parameter	*p*-Value	Hazard Ratio (95% CI)
Sex	0.645	1.145 (0.644–2.037)
Height	0.205	0.978 (0.945–1.012)
Body weight	0.225	0.988 (0.969–1.008)
BMI	0.468	0.979 (0.942–1.037)
Age	0.196	1.018 (0.991–1.045)
Preceded malignancy	0.383	1.364 (0.679–2.741)
Diabetes	0.785	1.092 (0.582–2.049)
Arterial hypertension	0.381	0.774 (0.435–1.374)
Hepatitis B	0.645	0.784 (0.278–2.209)
Hepatitis C	0.085	0.402 (0.143–1.133)
Alcohol abuse	0.971	0.963 (0.132–7.051)
PBC	0.709	0.683 (0.092–5.054)
PSC	0.564	1.800 (0.244–13.299)
CIBD	0.582	0.571 (0.078–4.199)
Gastritis	0.400	1.336 (0.680–2.626)
Lymphatic metastasis	0.574	1.246 (0.578–2.688)
Vascular metastasis	0.880	1.082 (0.388–3.022)
Pulmonary metastasis	0.704	0.872 (0.430–1.767)
Osseus metastasis	0.403	1.488 (0.587–3.773)
Suprarenal gland metastasis	0.618	0.602 (0.082–4.421)
**Peritoneal metastasis**	**0.003**	**0.372 (0.194–0.713)**
**Other metastasis**	**0.006**	**0.362 (0.174–0.752)**
Any metastasis	0.110	0.578 (0.295–1.132)
Sodium	0.127	0.926 (0.838–1.022)
Potassium	0.275	1.454 (0.742–2.849)
Calcium	0.230	0.287 (0.037–2.203)
Creatinine	0.649	0.979 (0.895–1.071)
GFR	0.198	0.992 (0.979–1.004)
Uric acid	0.784	1.033 (0.818–1.305)
Bilirubin	0.318	1.066 (0.940–1.209)
ALT	0.897	1.000 (0.997–1.003)
AST	0.938	1.000 (0.996–1.004)
γGT	0.603	1.000 (0.999–1.001)
**CRP**	**0.002**	**1.124 (1.044–1.211)**
Albumin	0.207	0.646 (0.328–1.274)
Hemoglobin	0.174	0.902 (0.778–1.046)
MCV	0.664	1.010 (0.967–1.054)
MCH	0.824	0.987 (0.880–1.107)
**Thrombocytes**	**0.030**	**1.003 (1.000–1.006)**
Quick	0.605	1.006 (0.983–1.029)
INR	0.841	0.918 (0.396–2.125)
aPTT	0.754	1.009 (0.955–1.066)
AFP	0.861	1.000 (1.000–1.000)
CEA	0.174	1.001 (0.999–1.004)
CA19-9	0.087	1.000 (1.000–1.000)
**L3SMI Cut-Off 71.95**	**0.020**	**1.990 (1.115–3.551)**
**L3PMI Cut-Off 6.345**	**0.037**	**2.384 (1.054–5.393)**
**MMA Cut-Off 30.51**	**0.011**	**2.176 (1.192–3.971)**

BMI: Body Mass Index, PBC: primary biliary cholangitis, PSC: primary sclerotic cholangitis, CIBD: chronic inflammatory bowel disease, GFR: glomerular filtration rate, ALT: alanine aminotransferase, ALT: aspartate aminotransferase, γGT: Gamma-glutamyltransferase, CRP: C-reactive protein, MCV: mean corpuscular volume, MCH: mean corpuscular hemoglobin, INR: international normalized ratio, aPTT: activated partial thromboplastin time, AFP: alpha-fetoprotein, CEA: carcinoembryonic antigen, CA19-9: carbohydrate antigen 19-9, L3SMI: skeletal muscle index at level L3, L3PMI: psoas muscle index at L3, MMA: muscle mass attenuation. Bold denotes statistical significance (*p* < 0.05).

**Table 3:** Multivariate Cox regression analysis for the prediction of overall survival.

Parameter	*p*-Value	Hazard Ratio (95% CI)
CRP	0.462	1.060 (0.908–1.236)
Thrombocytes	0.101	1.003 (0.999–1.007)
CEA	0.846	1.000 (0.997–1.003)
CA19-9	0.582	1.000 (1.000–1.000)
L3SMI Cut-Off 71.95	0.748	0.854 (0.326–2.237)
L3PMI Cut-Off 6.345	0.462	1.463 (0.530–4.038)
**MMA Cut-Off 30.51**	**0.035**	**2.264 (1.059–4.842)**

CRP: C-reactive protein, CEA: Carcinoembryonic antigen, CA19-9: Carbohydrate antigen 19-9, L3SMI: skeletal muscle index at level L3, L3PMI: psoas muscle index at L3, MMA: muscle mass attenuation. Bold denotes statistical significance (*p* < 0.05).

## Data Availability

Data are available upon request from the Department of Gastroenterology, Hepatology and Infectious Diseases of the University Hospital Düsseldorf for researchers who meet the criteria for access to confidential data: Wissenschaft.Gastro@med.uni-duesseldorf.de.

## Supplementary Materials

The following are available online at https://www.mdpi.com/article/10.3390/jcm10194340/s1, Figure S1: Overall survival is comparable between male and female CCA patients, Figure S2: Overall survival is comparable between CCA patients with or without distant metastases.
