# Supplementary figures and images for: Sarcopenia and Myosteatosis as Prognostic Markers in Patients with Advanced Cholangiocarcinoma Undergoing Palliative Treatment
Source: J Clin Med. 2021 Sep 23;10(19):4340. doi: 10.3390/jcm10194340 (PMC8509694; doi:10.3390/jcm10194340)

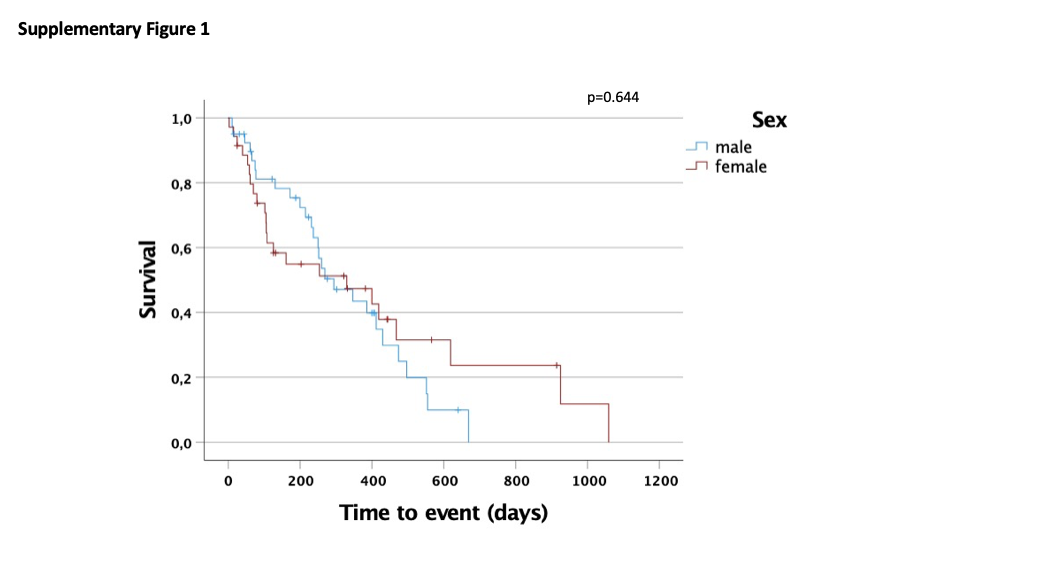

Supplement: Supplementary file 1 [file jcm-10-04340-s001.zip › Supplemental Figure1.png]

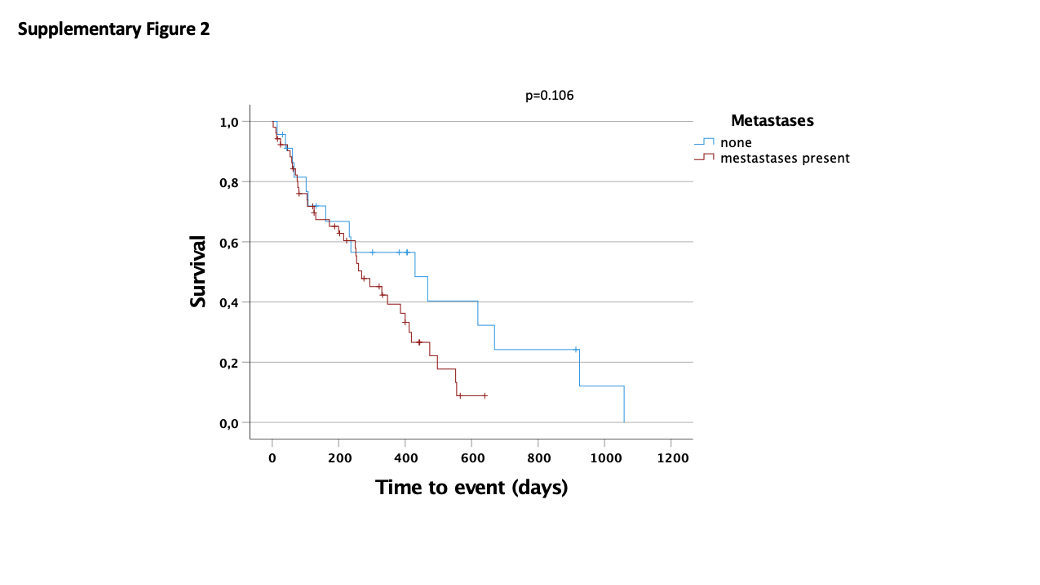

Supplement: Supplementary file 1 [file jcm-10-04340-s001.zip › Supplemental Figure2.png]
